# Inorganic Nanoparticles: Tools to Emphasize the Janus Face of Amphotericin B

**DOI:** 10.3390/antibiotics12101543

**Published:** 2023-10-15

**Authors:** Ariane Boudier, Nour Mammari, Emmanuel Lamouroux, Raphaël E. Duval

**Affiliations:** 1 Université de Lorraine, CITHEFOR, F-54000 Nancy, France; 2 Université de Lorraine, CNRS, L^2^CM, F-54000 Nancy, France; nour.mammari@univ-lorraine.fr (N.M.); emmanuel.lamouroux@univ-lorraine.fr (E.L.); 3 ABC Platform^®^, F-54505 Vandœuvre-lès-Nancy, France

**Keywords:** redox properties, oxidative stress, Amphotericin B, antimicrobial, inorganic nanomaterials

## Abstract

Amphotericin B is the oldest antifungal molecule which is still currently widely used in clinical practice, in particular for the treatment of invasive diseases, even though it is not devoid of side effects (particularly nephrotoxicity). Recently, its redox properties (i.e., both prooxidant and antioxidant) have been highlighted in the literature as mechanisms involved in both its activity and its toxicity. Interestingly, similar properties can be described for inorganic nanoparticles. In the first part of the present review, the redox properties of Amphotericin B and inorganic nanoparticles are discussed. Then, in the second part, inorganic nanoparticles as carriers of the drug are described. A special emphasis is given to their combined redox properties acting either as a prooxidant or as an antioxidant and their connection to the activity against pathogens (i.e., fungi, parasites, and yeasts) and to their toxicity. In a majority of the published studies, inorganic nanoparticles carrying Amphotericin B are described as having a synergistic activity directly related to the rupture of the redox homeostasis of the pathogen. Due to the unique properties of inorganic nanoparticles (e.g., magnetism, intrinsic anti-infectious properties, stimuli-triggered responses, etc.), these nanomaterials may represent a new generation of medicine that can synergistically enhance the antimicrobial properties of Amphotericin B.

## 1. Introduction

**Figure 1:**
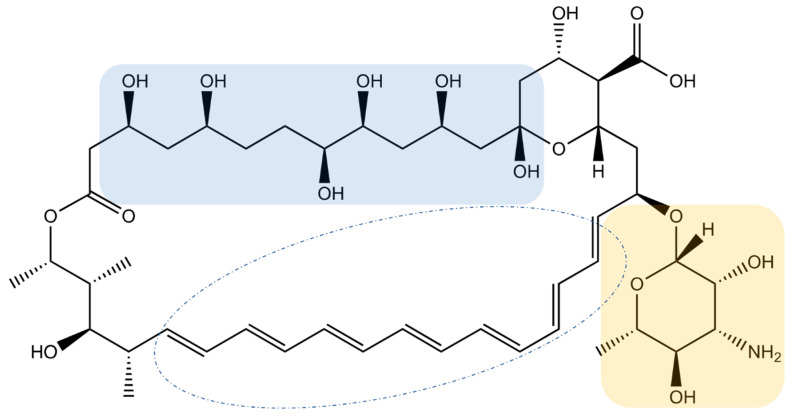
Molecular structure of AmB. Blue zone: polyol domain, yellow zone: deoxysugar mycosamine group.

Amphotericin B (AmB) is the leading compound of the polyene macrolide family, so named because of the numerous conjugated double bonds in a large macrolactone ring (Figure 1). Its structure also contains a polyol domain and a deoxysugar mycosamine group.

AmB is an old molecule as it was first discovered and extracted in the 1950s in Venezuela from *Streptomyces nodosus* [1,2]. The molecule rapidly reached the market after the FDA approved it in 1958 [2]. AmB is considered to have a broad spectrum of activity not only on fungi (i.e., filamentous, molds, yeasts, etc.) but also on parasites (e.g., Leishmania). Thus, AmB is efficient against different fungal genera/species: *Candida* spp., *Aspergillus* spp., *Histoplasma capsulatum*, *Coccidioides immitis*, *Blastomyces dermatitidis*, *Rhodotorula* spp., *Cryptococcus neoformans*, *Sporothrix schenkii*, *Fusarium* spp., *Cladosporium* spp., *Scytalidium* spp., and Zygomycetes. Conversely, the genera/species *Candida lusitaniae*, *Candida auris*, *Trichosporon* spp., *Geotrichum* spp., *Scedosporium* spp., *Fusarium* spp., and *Aspergillus terreus* are resistant or less sensitive to this molecule [3,4]. It should be noted that resistance to polyenes still remains rare (i.e., compared to resistance to other antifungal drugs, such as azoles). Furthermore, although several mechanisms of resistance to polyenes have been described in the literature, the main mechanism of resistance remains associated with a modification in the sterol composition at the level of the cell membrane or even a depletion of ergosterol, attributable to gene-level mutations involved in its biosynthesis [5,6]. Noteworthily, it is more and more common to find conflicting data regarding the activity of AmB against different fungal species/strains in the literature [4].

The affinity of AmB for the ergosterol of the membranes of microorganisms gives it its selective microbial activity. This selectivity is only slightly higher compared to that of cholesterol from mammalian cell membranes, making its therapeutic efficacy very narrow [4,5,6]. Considering the structure of the compound, studies demonstrated that the dimers forming AmB are toxic for eukaryotic cells. While the polyaggregated forms present reduced resistance for host cells, they retain antiparasitic activity at the same time [7]. AmB is mainly used in monotherapy, rarely as first-line, except for in the management of serious systemic fungal infections. AmB can also be used in combination with other antifungals such as flucytosine or fluconazole depending on particular clinical situations [8]. However, treatment with AmB is not devoid of side effects, which occur in 25 to 90% of patients [3,9]. The reported symptoms range from infusion-related reactions up to anaphylaxis, which can be prevented by drugs (e.g., corticoids, antihistamines, analgesics, etc.). Another serious side effect is a significant risk of nephrotoxicity which limits its use [10]. The formulation of AmB is an important topic of research with the aim to develop forms which improve its therapeutic effect and lead to less nephrotoxicity [11,12]. All the formulations are based on lipidic compounds mixed with AmB due to the amphiphilic nature of the antifungal. The lipid formulations of AmB which have been developed are either AmB in a colloidal dispersion, or AmB in a lipid complex or liposomal AmB. Thus, these three formulations differ in their lipid composition and therefore in their physical and pharmacokinetic characteristics, their efficacy, and their tolerance to efficacy [13]. Evidence has been shown that self-assembled mixed micelles containing AmB based on a combination of lecithin with polymers have reduced in vitro cytotoxicity and improved AmB solubility results with increased parenteral and oral bioavailability in rats compared to Fungizone^®^ [14]. Moreover, the oral administration of AmB encapsulated in nanoparticles (N-palmitoyl-N-methyl-N,N-dimethyl-N,N,N-trimethyl-6-O-glycol chitosan) has also showed high efficacy in mouse models of candidiasis, aspergillosis or visceral leishmaniasis compared to AmBisome^®^ administered parenterally [15]. Data have highlighted that using the lipid-based formulation of AmB is more expensive than conventional micellar deoxycholate AmB, which is why its use is limited in clinical practice [3,4]. This evidence has been discussed in numerous bibliographic reviews presenting the various formulations of AmB. Table S1 summarizes and compares the main content of these studies, and these will not be emphasized in the present paper. The development of an orally active formulation of AmB capable of reducing the systemic drug toxicity, avoiding infusion-related adverse events, improving patient compliance, and reducing the costs associated with the intravenous administration of commercial formulations of AmB is an urgent requirement. Up until now, in contrast to lipid formulations, no inorganic nanoparticles, as an agent to carry AmB, have been brought to clinical trials. However, they have unique specific properties (such as magnetic, optical, redox, etc.) that can be added to those of AmB or beneficially influence those of AmB synergistically. Moreover, there are many reports of the pre-clinical development of such objects as carriers of AmB or for the development other anti-infectious strategies [16]. Inorganic nanoparticles are structured with a well-organized core made of metal or carbon atoms surrounded by an organic corona (i.e., inorganic/organic core–shell particles). The core can bring, depending on the material it is made with, magnetic or optical properties, while the corona can be functionalized by drugs (e.g., AmB) or other molecules. They represent ideal tools for the targeting and the recognition of the site of action (antibody, aptamer, substrate for an enzyme, ligand for a receptor) [17]. They can be combined with other properties (i.e., interact with radiation) to succeed in the development of nano-objects dedicated for both therapy and diagnostics (i.e., theranostic) [17,18]. These nanoparticles often exhibit redox properties. This is very interesting since it has now also been described that AmB is characterized by both oxidant and reductive activities, usually known as a Janus face. Indeed, Janus was a Roman god depicted with two faces; one looking ahead, the other behind.

In this review, in the first step, the similarities between inorganic nanoparticles and AmB will be emphasized according to their redox character. In the second step, the different strategies to synthesize inorganic nanoparticles as AmB carriers will be discussed. A specific emphasis will be given to the capacity of inorganic nanoparticles to enhance either the prooxidant or the antioxidant effects of AmB. An explanation of the involved mechanisms and the synergistic anti-infectious effects will be described, which represents the originality of the present review.

## 2. Similarities in Redox Behaviors between Amphotericin B and Inorganic Nanoparticles

### 2.1. Redox Properties of Amphotericin B

#### 2.1.1. The Janus Face of Amphotericin B

**Figure 2:**
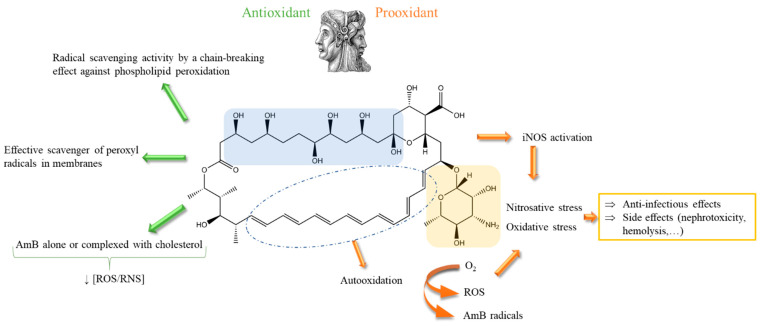
Janus face of AmB: prooxidant and antioxidant effects described in [5,21,22,23]. ROS: reactive oxygen species; RNS: reactive nitrogen species; iNOS: inducible nitric oxide synthase.

AmB possesses a double role, either as a prooxidant or, in some publications, as an antioxidant. The main mechanisms for this are illustrated in Figure 2. This redox role is implied in the mechanism of anti-infectious action, as well as other (such as polyene-sterol: ergosterol) interactions leading to membrane destabilization with pore formation and/or surface adsorption and/or the formation of sterol aggregates (or “sponges”) outside the cell membrane causing the loss of ions and the accumulation of reactive oxygen species (ROS) [5,19,20].

The prooxidant effect of AmB is quite significantly described in the literature. It leads to both oxidative and nitrosative stresses through the expression of stress genes, including an increase in the inducible form of nitric oxide synthase, the generation of ROS and reactive nitrogen species (RNS), respectively, and the production of proinflammatory cytokines [5,20,21]. Radicals originating from AmB itself were also identified using electron-spin resonance (ESR) spectroscopy [24]. Oxidative and nitrosative stresses damage the plasma membrane, the intracellular proteins, the mitochondria activity, and the nucleic acids [5]. AmB has been shown to have the ability to trigger a common dependent cell death pathway through oxidative damage in fungi such as *Candida albicans*, *Saccharomyces cerevisiae* or *Cryptococcus gattii* via the production of ROS in a tricarboxylic acid cycle and respiratory chain-dependent manner impacting, consequently, the inhibition of its DNA repair systems [20,25,26,27].

It is worth underlining that the antioxidant property of AmB is less described in the literature. The antioxidant effect may originate from the polyol part of the molecule [21]. It was evidenced in vitro [22] and was also highlighted in rat aortic smooth muscle cells [23]. This dual behavior has already been described for other molecules such as retinol [28], ascorbic acid [29] or Trolox [30]. As a function of the imposed conditions, a conversion from one property of AmB to the other (i.e., pro- to anti-oxidant and vice versa) may occur. The questions of how important the antioxidant phenomenon is, or how the equilibrium between the prooxidant and antioxidant properties of AmB is balanced, for the activity and/or toxicity of AmB, remain unsolved. These issues are extremely difficult to address due to the intimate interdependence of this phenomenon.

#### 2.1.2. Amphotericin B Activity, Resistance, and Toxicity, and Its Possible Modulation

The impact of both oxidative and nitrosative stresses are well-described for AmB activity in fungi (i.e., filamentous, molds, yeasts, etc.) and parasites [5,26,31], as well as for AmB-resistant pathogens [5,31,32,33]. These elements were deeply reviewed by Carolus and coll. recently [5]. One less-studied aspect in the literature is the impact of oxidative and nitrosative stresses on AmB-induced toxicity. These stresses are identified as actors in the induced side effects of AmB in clinical settings, on kidney and liver [34,35,36,37]. Its dose-dependent toxicity is caused by ROS (and maybe also by RNS, even if this is usually less studied) and the oxidized forms of AmB.

Because of the consequences implied by oxidative and nitrosative stresses on AmB activity, resistance, and toxicity, the modulation of redox status by the co-administration of other oxidants or antioxidants with AmB has been considered by researchers with the aim of the enhancement of its anti-infectious property and/or limitations of its toxicity [5]. A great variety of components were shown to enhance AmB activity when co-administered with redox-potent molecules. For example, Kim and coll. showed that thymol enhances AmB activity on *Candida albicans* and *Candida krusei* [38]. They also demonstrated that dihydroxybenzaldehydes promote AmB activity against *C. albicans*, *C. krusei*, *C. tropicalis*, and *Cryptococcus neoformans* [39]. One can also mention the effect of butylated hydroxyanisole, *n*-propylgallate, or nordihydroguaiaretic acid on *C. albicans* and *C. parapsilosis* [40], and ascorbic acid on *Aspergillus terreus* [41]. The four involved mechanisms were (i) the co-disruption of the redox signaling on the response capacity of pathogens [39], (ii) the targeting of at least one common cellular component in the antioxidant system of the organism [39], (iii) a prolonged duration of AmB activity via a stabilizing effect probably preventing its auto-oxidation and stabilizing the polyene moiety of the molecule [39,40], and (iv) a synergistic prooxidant effect, increasing the concentration of ROS, lowering the minimal inhibitory concentration (MIC) and restoring the sensitive phenotype of a AmB-resistant strain [41].

On the contrary, well-known antioxidants failed to induce any anti-infectious activity. *N*-acetylcysteine (a precursor of glutathione) improved the survival of *A. fumigatus* in the presence of AmB [42]. This molecule also showed a protective effect against ROS induced by AmB in *Aspergillus terreus* [41]. Similarly, the addition of reduced glutathione or cysteine revived the endospores of *Coccidioides immitis* previously treated with AmB by modulating the redox potential of the medium [43]. In parallel, cysteine stopped the AmB-mediated growth inhibition of *C. albicans* [44]. 

Several redox-balancing agents were also tested to counterbalance the toxicity induced by AmB. For example, pre-treatment with diosmin hesperidin in Wistar rats followed by AmB administration showed an antioxidant protective effect on the kidneys [36]. In another study, the co-administration of vitamins A and E with AmB attenuated the side effect of the antifungal on the kidneys and liver of Wistar rats. The combination of both vitamins was more efficient than each vitamin alone [37]. Another antioxidant, caffeic acid phenethyl ester, showed an effectiveness as an adjuvant agent for AmB nephrotoxicity in rat models [34].

In addition, the mechanisms of AmB resistance of certain clinical isolates such as the *Candida haemulonii* species complex (*C. haemulonii*, *C. duobushaemulonii*, *C. haemulonii* var. *vulnera*) have been explored. Consequently, studies on the molecular composition of the wall in this group of fungi revealed that the vast majority of the membrane sterols were intermediates of the ergosterol pathway, and not ergosterol itself, highlighting the absence of an AmB target and thus explaining (at least in part) the resistance phenotype [45]. These results were supported by the fact that the deletion of the genes encoding ergosterol (*ERG11* and *ERG3* genes; encoding lanosterol 14-demethylase and C-5-sterin desaturase, respectively) affect the resistance of *C. lusitaniae* and of *Saccharomyces cerevisiae* strains [46,47,48]. Thus, a decrease in sterol (i.e., ergosterol) content causes a decrease in the membrane permeability to the compound [45]. The majority of studies have shown that AmB induces the formation of ROS as described above; however, this phenomenon was slightly observed in the strains of the *C. haemulonii* species complex. Evidence has determined that these fungal strains have undergone an alteration of the respiratory chain: poor growth in unfermented carbon sources, low oxygen consumption, and an alteration of mitochondrial membrane potential. These data explain the resistance presented in this multi-resistant fungal complex with respect to AmB [45].

### 2.2. Redox Properties of Inorganic Nanoparticles

**Figure 3:**
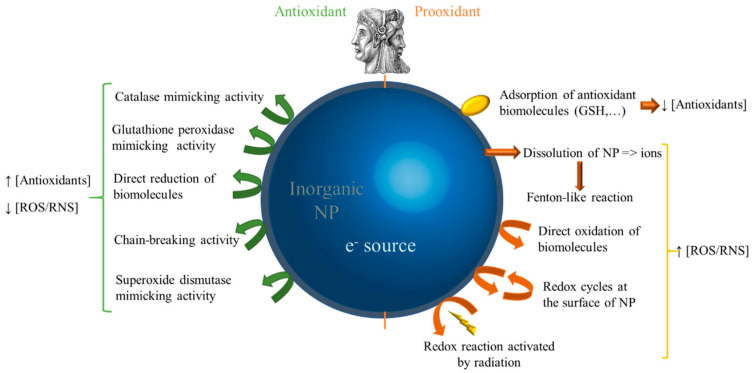
Summary of the main mechanisms conferring antioxidant or prooxidant properties to inorganic nanoparticles. ROS: reactive oxygen species; RNS: reactive nitrogen species; GSH: reduced glutathione; NP: nanoparticle.

There are important similarities between the behaviors of AmB and inorganic nanoparticles as they are both characterized by prooxidant and antioxidant properties. Inorganic nanoparticles behave as redox-potent agents using an important variety of mechanisms which are depicted in Figure 3. For a detailed view of this chemistry, the reader is referred to the following reviews [48,49,50,51,52,53].

Nanoparticles interfere with the redox homeostasis of a medium via different pathways: either directly, e.g., by providing electrons for the direct self-conversion of an antioxidant to a prooxidant molecule and vice versa [54], or indirectly, e.g., by the nanoparticle degradation via dissolution [55] or upon radiation [56].

The redox properties of nanoparticles are highly dependent on the type of material that they are made of (e.g., carbon, metal, metallic oxide, etc.) [48,50], the process by which they were prepared [56], the shape and their isotropy/anisotropy [57], their capping in terms of type/force of interaction, and the nature of the capped molecule [58,59]. The redox potential of nanoparticles or their oxidative potential (prooxidant character) remains difficult to assess because of the low concentrations of, for example, synthesized nanoparticles, possible interference with the analytical method, and the relevance of the incubation medium [48,49]. In parallel to what was explained as the case for AmB, the redox properties of inorganic nanoparticles are involved in their related activity in cells and in their induced toxicity, even if this last point is a matter of debate in the literature [49]. The possible biological impact of this double-faced redox property is observed on the cell wall and membrane, on the proteins, and on the nucleic acids [60]. The overall response of the organism is either a regulation of redox homeostasis via redox signaling or stress that can lead to necrosis, apoptosis, autophagy, etc. [49]. This prooxidant effect has been used as a bacterial-killing agent, which is now being explored for further use in antimicrobial medicine for the treatment of infections due to multi-resistant bacteria [60]. Various studies have been able to highlight the antibacterial activity of silver nanoparticles (AgNPs) [61,62,63]. These metallic nanoparticles promote the induction of ROS leading to structural and metabolic damage which ultimately leads to an antibacterial effect [64]. Of note, an extensive review about the oxidative-stress-mediated antimicrobial properties of metal-based nanoparticles has been recently published [60].

Some inorganic nanoparticles are functionalized by redox-potent molecules in order to obtain synergy in their antioxidant activity. These tools are sometimes named “nanoantioxidants” [65]. Many types of nanoparticles have been functionalized with different antioxidants. The main results were a prolonged release of the antioxidant, an improved biocompatibility, and a targeted delivery of the antioxidants with superior antioxidant profiles [65].

## 3. Inorganic Nanoparticles Carrying Amphotericin B

### 3.1. The State-of-the-Art of Lipidic Formulations of Amphotericin B on the Market or under Clinical Trials

Due to its chemical structure, AmB is lipophilic, completely insoluble in water, sparingly soluble in alcohol, and highly soluble in dimethylformamide or dimethylsulfoxide [66]. Even though the molecule presents two groups (carboxylic acid and primary amine) associated with ionization constants (pKa), the molecule is globally neutral at physiological pH as it is both positively and negatively charged. AmB is characterized by poor oral permeability, besides a degradation occurring in the stomach. AmB is presented in its classical formulation as micelles of sodium deoxycholate. These parameters may explain why researchers focus on its formulation in so many works. The nanoparticle formulations based on liposomes or lipids increase the therapeutic index of the molecule, decreasing its toxicity, especially nephrotoxicity, while retaining the same efficacy [67,68,69]. Indeed, lipid formulations of AmB limit nephrotoxicity, but tubule cells remain still vulnerable to some forms of superimposed injury [70]. In 2020, Hnik and coll. tested a single dose of an oral formulation based on liposomal amphotericin (iCo-019) on healthy people. The objective of this study was to develop a molecule that is easy to administer, stable, and non-toxic while maintaining effective pharmacological activity. The data of the randomized controlled trial has demonstrated that the single dose of iCo-019 demonstrated a good tolerance of the molecule and a reduction in its toxicity [71]. More precisely, an overview of the formulations on the market or under clinical trial is presented in Table 1.

These formulations present an innovation, particularly in limiting nephrotoxicity, which explains why they are reserved to treat people suffering from kidney diseases. The products under clinical trial clearly open new opportunities in terms of administration routes. However, from a redox point of view, they do not present any of these properties.

### 3.2. Inorganic Nanoparticles as Modulator of AmB Redox Properties

#### 3.2.1. Strategies to Functionalize Inorganic Nanoparticles with Amphotericin B

Numerous nanoparticles were synthesized and functionalized to obtain particles carrying AmB. Table 2 presents an overview of the published works.

**Figure 4:**
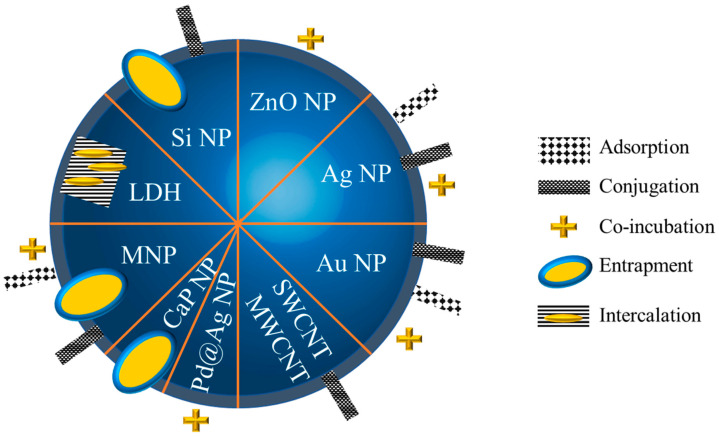
Different strategies employed to carry AmB. NP: nanoparticles; LDH: layered double hydroxide; MNP: magnetic nanoparticles; CaPNP: calcium phosphate nanoparticles; SWCNT: single-walled carbon nanotubes; MWCNT: multi-walled carbon nanotubes.

The nanoparticles were made of a metal or metallic oxide (e.g., silver, gold, iron, and zinc), or they were based on carbon (with carbon quantum dots, graphene, nanotubes) or on calcium phosphate, or on layered double hydroxides, or on silica, or even based on core–shell particles (Pd@Ag nanoparticles) [75,83,100,101]. The synthesis of nanoparticles was realized mainly via the bottom-up approach (using building blocks that further organize in nanoparticles upon a trigger, e.g., reduction, irradiation, etc.). A majority of researchers used chemical processes, while some research described the production of nanoparticles (Ag, Au and iron oxide) via different methods: phytosynthesis using extracts of *Isatis tinctoria*, *Maytenus royleanus* [75], *Cucumis melo L var makuwa*, *Prunus persica* L. [85], using Chinese cabbage or maize silky hair [102]; or using a green synthesis by *Punica granatum* [103]; or by biosynthesis using *Acidophilic Acinetobacter P. columellifera* subsp. *Pallida* [76]; or 14 *Acinetobacter* spp. isolates [77]. In addition, two studies used AmB to directly reduce the Ag^+^ into Ag^0^ or Au^3+^ into Au^0^ with success, highlighting the antioxidant character of AmB [78,83]. The strategies used to obtain nanoparticles carrying AmB are illustrated in Figure 4.

Common strategies were developed to carry the drug: they rely on adsorption, i.e., a weak interaction between the silver core and mycosamine group or polyol group [78,104] or between the nanoparticle and AmB; or conjugation, realized by a strong interaction, e.g., covalence with the use of a spacer [84,88]; or entrapment or intercalation between layers [105] within the nanoparticle and the simple co-incubation of nanoparticles and AmB.

In some studies, the authors took advantage of the unique properties of the inorganic nanoparticles, besides their capacity to modulate the redox signaling of the organisms (see Section 3.2.2 and Section 3.2.3). For example, Ahmad and coll. demonstrated an increase in the activity of their silver nanoparticles carrying AmB upon UV irradiation [73]. AgNP are particularly studied because they have also been known for years for their anti-infectious activity as explained above. In another study, carbon quantum dots were functionalized by AmB and used as a new method for the specific detection of *C. albicans* for diagnostic purposes [106]. Iron oxide nanoparticles are also interesting due to their response to a magnetic field that can induce the generation of controlled non-invasive heat and efficient drug delivery at the selected site [85]. Various designs of iron oxide nanoparticles (34–40 nm) coated with bovine serum albumin and targeted with AmB (AmB-IONP), were formulated via a layer-by-layer approach, and tested for their antifungal activity. These compounds showed improved antifungal activity efficacy against *C. albicans* and *C. glabrata* clinical isolates [97]. There are numerous works developed in that sense (Table 2).

#### 3.2.2. Inorganic Nanoparticles as Synergic Prooxidants

**Figure 5:**
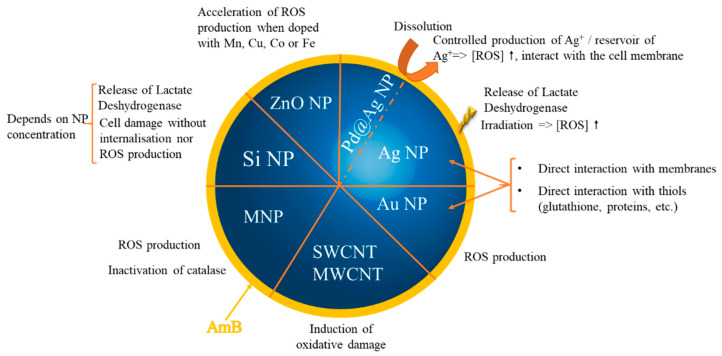
Main mechanisms implied in the prooxidative effect of nanoparticles carrying AmB at the origin of the described synergistic activity. NP: nanoparticles; MNP: magnetic nanoparticles; SWCNT: single-walled carbon nanotubes; MWCNT: multi-walled carbon nanotubes; ROS: reactive oxygen species.

Among the published articles, a lot of studies highlight the combined or synergistic redox properties of nanoparticles carrying AmB. Only a few papers concentrated on their activity against pathogens without exploring the involved redox mechanisms. The proposed redox mechanisms are represented in Figure 5. One can easily understand that oxidative stress can be generated by nanoparticles and/or AmB and then self-sustained. It is very difficult to determine the first actor due to the tight interconnectivity of the mechanisms.

A synergistic effect was almost always highlighted when an oxidative stress was either demonstrated or hypothesized. The effect is therefore superior to the one induced by nanoparticles or AmB alone. Recently, the same phenomenon was observed with AmB and gentamicin-loaded nanosheets/nanoneedles-based boron nitride films [107]. These films exerted an anti-infectious activity against *Neurospora crassa* and antibiotic-resistant *E. coli*. Another study using molecules other than AmB also showed the synergic effect of nanoparticles carrying antibiotics explained by oxidative stress, for example, silver nanoparticles combined with ampicillin, chloramphenicol, and kanamycin [108] or with neomycin or gentamicin [109]. However, besides their redox properties, nanoparticles possess other advantages, since they can pass through physiological barriers and penetrate more easily into pathogens due to their small size [77,110]. After entering into the cells, the nanoparticles disrupt the membrane integrity which creates a passage for drugs across the cell membrane, improving their action at the target site. This was shown for silver nanoparticles [77]. Amphotericin B-silver hybrid nanoparticles (AmB-Ag) have been reported to be a highly effective form of this antibiotic to combat fungi. In a study analyzing the interaction of AmB-Ag with *C. albicans* cells using molecular spectroscopy and imaging techniques, the antifungal activity of the nanocomplex system of the disintegration of the cell membrane was demonstrated, which occurs within a few minutes of treatment. This activity increases considerably when the treatment is in the form of hybrid silver nanoparticles. Experimental results show that AmB-Ag can effectively cross the cell wall barrier and deliver antibiotic molecules to cell membranes, thus activating oxidative stress [111].

Nevertheless, this prooxidant effect was sometimes the origin of a toxicity [112]. Researchers have demonstrated that the toxicity of silica nanoparticles carrying AmB was more important than that of the unloaded silica nanoparticles on human fibroblasts and on human endothelial cells. Moreover, the same authors have demonstrated that amphotericin B-functionalized SiO_2_ NPs with an average size of 5 and 80 nm have antifungal activity against several strains of *Candida* species [113]. This effectiveness was also demonstrated when SiO_2_ NPs were immobilized using amphotericin B in the case of dental resins [114]. In another study, AmB macrocyclic polyene was used as a reducing agent and stabilizing agent during the manufacture of Ag NPs. AmB-Ag nanoparticles (with an average size of 4 nm) have an inhibitory effect on the growth of *Aspergillus niger*, *Candida albicans*, and *Fusarium culmorum*. The authors attributed the high antifungal effectiveness of AmB-Ag NPs to the synergistic effect between AmB and Ag^+^ ions [78].

ZnO-PEGylated AMB (ZnO-AmB-PEG) nanoparticles demonstrated their antifungal effects on two strains of *Candida* spp. When comparing the results obtained by treatment with ZnO-AmB NPs and free AMB against *C. albicans* and *C. neoformans*, it was determined that ZnO-AmB-PEG NPs significantly reduced the growth of fungi. Additionally, the toxicity was studied using in vitro blood hemolysis, in vivo nephrotoxicity. ZnO-AmB-PEG significantly reduced leukocyte counts, creatinine, and blood urea nitrogen levels, compared to AmB. The authors suggested that ZnO-AmB-PEG could be tested and used clinically [115]. On the contrary, other works reported an absence of toxicity on the kidneys, liver, and spleen of Golden Syrian hamsters [87], Swiss mice [91] and Balb/c mice [95] as well as on red blood cells [79]. In the latter, this was explained by the association of the functionalized nanoparticles with the circulating high-density (HDL) and low-density lipoproteins (LDL). Toxicity issues related to inorganic nanoparticles are a long-running story. Among others, the physicochemical parameters of nanoparticles, the material they are made with, and their possible degradation products are key points to understand since they may explain the observed phenomenon. It remains very difficult to express general rules about this toxicity [61,116].

#### 3.2.3. Inorganic Nanoparticles as Synergic Antioxidants

Two publications focused on the antioxidant activity of nanoparticles carrying AmB [85,102]. In both, nanoparticles (made either of magnetite iron oxide or of gold) were synthesized using plants: either the silky hair of corns or the outer leaves of Chinese cabbage or other aqueous extracts of outer oriental melon peel and peach. It is likely that the nanoparticle corona contained antioxidant biomolecules such as flavonoids and polyphenols besides the activity of the metallic core of the nanoparticles. In the two works, the authors highlighted a strong antioxidant property due to the scavenging of radicals (i.e., 1,1-diphenyl-2-picrylhydrazyl, 2,2′-azino-bis(3-ethylbenzothiazoline-6-sulphonic acid) and nitric oxide) and also a strong proteasome inhibition. It has already been described that the antioxidant activity coming from the inorganic core of nanoparticles can be enhanced when functionalized by other antioxidants such as reduced glutathione [117]. These nanoparticles, when combined with AmB, proved to have synergic activity against *Candida* spp. The level of antioxidant property was correlated to the antifungal activity.

The synergic antioxidant effect is less studied in the literature. The obtained antioxidant effect may be linked to the corona of such nanoparticles that are based on extracts of plants, which can bring an antioxidant activity by themselves. The synergistic aspect of the nanoparticle combined with AmB is not totally obvious in these examples. Other studies will certainly bring more robustness to this activity in the future.

## 4. Summary and Future Directions

Both AmB and inorganic nanoparticles exhibit a Janus face through their redox activities. The first generation of formulations is already on the market and is based on lipids. In this review, a second generation of nanoparticles carrying AmB was reviewed to highlight their capacity to behave as synergic prooxidants or antioxidants enhancing the redox properties of the molecule, and, as a consequence, increasing the therapeutic activity of AmB. Due to the unique properties of the inorganic nanoparticle, the pre-clinical development of objects carrying AmB will certainly be dedicated to the development of agents for theranostic (e.g., using light responsive nanoparticles) and/or for targeted delivery (e.g., using magnetic nanoparticles with the application of a magnetic field on the desired site). Indeed, one can easily imagine core corona nanoparticles (or even core multi-corona nanoparticles) combining the different advantages of their materials. For example, nanoparticles made with an iron oxide core for magnetic properties surrounded by a silver corona for their anti-infectious properties and, used for both, and their capacity to respond to UV-vis radiation to generate oxidative stress at the targeted site. The functionalization of such objects via AmB would be of great potential for precision therapy.

The future steps for such objects to reach the clinical level remain challenging: requiring proof of non-toxicity as well as non-immunogenicity (no adverse reaction, no accumulation in organs, etc.), and of their benefit vs. other therapies, provided that the industrial translation (e.g., scale-up, long-term stability) is feasible.

## Figures and Tables

**Table 1:** Formulations of AmB on the market or under clinical trial (clinicaltrials.gov (accessed on 15 September 2023).

AmB Formulation (Examples)	Administration Route	Market Level/Clinical Trial	Cost (in USD) *	Reference and/or Clinicaltrials.gov Number
Micelles of sodium deoxycholate (Fungizone^®^)	Intravenous	Registered in 1966 (FDA)	96	
Unilamellar liposomes (AmBisome^®^)	Intravenous	Registered in 1997 (FDA)	1646	
Ribbon-like lipid complexes (Ablecet^®^)	Intravenous	Registered in 1995 (FDA)	840	
Disc-shaped liposome (Amphocil^®^ or Amphotec^®^)	Intravenous	Registered in 1996 (FDA)	448	
Liposomal Amphotericin B	Intravenous	Clinical trial	-	NCT03529617 NCT05108545 NCT02025491 NCT05814432 NCT01122771 NCT00003938
Amphotericin-B	Intravenous	Clinical trial	-	NCT02283905 NCT00001017 NCT00002277
Amphotericin B Lipid Complex	Intravenous	Clinical trial	-	NCT00002019
Encochleated Amphotericin B	Oral	Clinical trial	-	NCT03196921 NCT05541107
Liposomal Amphotericin B gel 0.4%	Topical	Clinical trial	-	NCT02656797
Lipo-AB^®^ (Amphotericin B) liposome	Intravenous	Clinical trial	-	NCT03511820
Liposomal Amphotericin B (AmBisome^®^)	Intravenous	Clinical trial	-	NCT02320604 NCT00628719 NCT00418951 NCT00936910 NCT00362544
Liposomal Amphotericin B Amphotericin B deoxycholate	Single infusion	Clinical trial	-	NCT00628719
Liposomal Amphotericin B (AmBisome^®^)	Intravenous	Clinical trial	-	NCT00467883
Amphotericin B Lipid emulsion (Amphomul^®^) Liposomal Amphotericin B	Single infusion	Clinical trial	-	NCT00876824
Cochleated nanoparticles	Oral	Clinical trial	-	NCT02629419 [72]
Amphotericin B Cream 3%	Topical	Clinical trial	-	NCT01845727
Nebulized liposomes (AmBisome^®^)	Pulmonary	Clinical trial	-	NCT00177710 NCT00263315
		Clinical trial	-	NCT04502381 NCT00263315 NCT02273661
		Clinical trial	-	NCT04267497
Nebulized lipid complexes (Abelcet^®^)	Pulmonary	Clinical trial	-	NCT00177684 NCT00235651
	Intravenous	Clinical trial	-	NCT04225195
Nebulized AmB deoxycholate	Pulmonary	Clinical trial	-	NCT01857479
Nebulized Amphotericin B lipid complex	Pulmonary	Clinical trial	-	NCT01615809
Liposomal AmB	Intrathecal	Clinical trial	-	NCT02686853
Liposomal AmB	Oral	Clinical trial	-	NCT04059770

* per day for a 70 kg patient with the upper-limit dose.

**Table 2:** Results obtained from inorganic nanoparticles as AmB carriers.

Type of Nanoparticle	Core (dc) and Hydrodynamic (Dh) Diameter	Targeted Microorganism	Main Conclusion	References
Ag	dc = 10 nm to 15–20 nm (TEM) Dh = 8–15 nm to 15–25 nm (DLS)	*Leishmania tropica*	Synergic effect of nanoparticles and AmB Prooxidant effect	[73]
	Dh = 10–90 nm (AFM)	*Naegleria fowleri*	Synergic effect of nanoparticles and AmB Prooxidant effect	[74]
	dc = 8–15 nm (TEM) Dh = 10–17 nm (DLS)	*C. albicans* *C. tropicalis*	Synergic effect of nanoparticles and AmB Prooxidant effect	[75]
	dc = 12.7 nm (SEM)	*Malassezia furfur* *C. albicans* *Trichophyton erinacei*	Synergic effect of nanoparticles and AmB Prooxidant effect	[76]
	dc = 10–18 nm (TEM)	*C. albicans*	Synergic effect of nanoparticles and AmB even on biofilms Prooxidant effect	[77]
	dc = 7–15 nm (TEM) Dh = 11–17 nm (DLS)	*C. albicans* *A. niger* *Fusarium culmorum*	Synergic effect of nanoparticles and AmB No redox property studied	[78]
	Dh = 170 nm (DLS)	*P. aeruginosa* *C. albicans*	Effect on bacteria and on fungi No redox property studied	[79]
Ag	Dh = 30 nm (DLS)	*Resistant clinical isolates* *C. glabrata*	Effect on fungi No redox property studied	[80]
Ag	Dh = 18–60 nm (DLS)	*C. albicans* *C. tropicalis* *C. krusei* *C. parapsilosis* *C. glabrata*	Effect on fungi No redox property studied	[81]
Pd@Ag nanosheets	Hexagonal shape; dc = 11 nm, 30 nm, 80 nm, and 120 nm (TEM) with Ag/Pd ratio = 6 (ICP-MS)	*C. neoformans* *C. gattii*, *C. albicans* *C. glabrata* *C. krusei* *C. tropicalis* *C. parapsilosis* *A. fumigatus* *Rhizopus oryzae*	Synergistic fungicidal effect with AmB. More susceptibility for *Cryptococcus* spp. and *C. glabrata* whereas *R. oryzae* was insensitive Prooxidant effect	[82]
Au	dc = 50–200 nm (AFM)	*Ancathamoeba castellanii*	Increased bioactivity No redox property studied	[83]
	Dh = 50 nm (DLS)	*C. albicans*	Slightly more effective than bare AgNP Prooxidant effect	[84]
	Estimated absolute crystallite size = 40 and 78 nm (XRD)	*C. albicans* (2 strains) *C. glabrata* *C. geochares* *C. saitoana*	Synergic effect of nanoparticles and AmB Antioxidant effect	[85]
	Dh = 10–15 nm (DLS)	Resistant clinical isolates *C. glabrata*	Effect on fungi No redox property studied	[80]
	dc = 38.5 ± 10.6 nm (TEM)	*Aspergillus niger* *A. flavus* *A. fumigatus* *A. terreus*	Effect on fungi No redox property studied	[86]
Carbon	Graphene–carbon nanotubes composite	*Leishmania donovani*	Synergic effect of nanoparticles and AmB No redox property studied	[87]
	Ammonium functionalized multi- and single-walled carbon nanotubes dc = 140–500 to 1500–4000 nm (TEM)	*C. parapsilosis* *C. albicans * *C. neoformans*	Increase effect of nanoparticles and AmB No redox property studied	[88]
	Ammonium functionalized multi- and single-walled carbon nanotubes dc = 140–500 × 1500–4000 nm (TEM)	*C. neoformans and acapsular mutants* *Rhodotorula rubra* *S. cerevisiae* *Pichia etchellsii* *C. albicans* *C. parapsilosis*	Activity even against AmB-resistant strains Redox mechanisms hypothesized	[89]
	Functionalized carbon nanotubes dc = 40–70 nm × 2–8 µm (TEM)	*L. donovani*	Superiority over AmB in terms of toxicity and efficacy No redox property studied	[90]
Ca_3_(PO_4_)_2_	Dh = 112–165 nm (DLS)	*L. donovani*	More efficient to treat intracellular leishmania No redox property studied	[91]
Fe	dc = 13 nm (TEM)	*Candida* spp. *C. glabrata* *C. albicans*	Synergic effect of nanoparticles and AmB even on biofilm Prooxidant effect	[92]
	Dh = 184 nm (DLS)	*A. castellanii*	Synergic effect on trophozoites and on cysts No redox property studied	[93]
	dc = 10 nm (TEM) Dh = 15 nm (DLS)	*L. donovani*	Synergic effect of nanoparticles and AmB No redox property studied	[94]
	dc = 6–7 nm (TEM) Dh = 85 nm (DLS)	*P. brasiliensis*	Similar activity No redox property studied	[95]
	Sub-micronic particles (SEM)	*C. albicans* *C. glabrata* *C. geochares* *C. saitoana*	Synergic effect of nanoparticles and AmB Antioxidant effect	[85]
	Dh = 193–218 nm (DLS)	*A. castellanii*	Synergic effect of nanoparticles and AmB No redox property studied	[96]
	Dh = 30–40 nm (DLS)	*C. albicans* *C. glabrata* *C. krusei* *C. parapsilosis* *C. tropicalis*	time-dependent cellular uptake in *C. albicans* and *C. glabrata* clinical isolates, and improved efficacy over conventional AmB No redox property studied	[97]
Silica	Mesoporous included in a resin dc = 85 nm (TEM)	*C. albicans* *Streptococcus oralis*	Long-term effect of nanoparticles and AmB No redox property studied	[98]
ZnO	Doped with Fe or Mn or Co or Cu Not indicated	*C. neoformans* *Trichophyton mentagrophytes*	Synergic effect of nanoparticles and AmB mostly when doped Prooxidant effect	[99]
	dc = 10–30 nm (SEM)	*C. albicans* *C. tropicalis* *C. krusei* *C. parapsilosis* *C. lusitaniae*	Effect on fungi No redox property studied	[100]
Se	Dh = 105–209 nm (DLS)	Resistant clinical isolates *C. glabrata*	Effect on fungi No redox property studied	[80]
TiO_2_	dc = 10–25 nm (SEM)	*C. albicans* *C. tropicalis* *C. krusei* *C. parapsilosis* *C. lusitaniae*	Effect on fungi No redox property studied	[100]

## Data Availability

Not applicable.

## Supplementary Materials

The following supporting information can be downloaded at: https://www.mdpi.com/article/10.3390/antibiotics12101543/s1, Table S1. References [67,68,118,119,120,121,122,123,124] are cited in the supplementary materials.
