# Supplementary material for: Inorganic Nanoparticles: Tools to Emphasize the Janus Face of Amphotericin B
Source: Antibiotics (Basel). 2023 Oct 15;12(10):1543. doi: 10.3390/antibiotics12101543 (PMC10604816; doi:10.3390/antibiotics12101543)
Supplement: Supplementary file 1 [file antibiotics-12-01543-s001.zip › antibiotics-2656516-SI.pdf]

Supplementary data S1

Reference<br>Original points	Formulations			Drugs		Overview of	
	Liposomes	Lipid-based	Other nanoparticles or assimilated	AmB	Others	Products on the market	Products under clinical trials
Hafner et al., 2014.	x	x	x	x	x	x	
Discussion on nanosimilars, problems about the regulatory issues and the translation from bench to market							
Bekersky et al., 1999.	x	x		x			
Focus on the clinical data of AmB formulations							
Allen and Cullis, 2013.	x			x	x	x	x
Discussion on PEGylated liposomes, triggered release liposomes, targeted liposomes							
Bobo et al., 2016.	x		x	x	x	x	x
Chronologic overview of the market							

Anselmo and Mitragotri, 2016.<br>Anselmo and Mitragotri, 2019.	x		x	x	x	x	x
Discussion on translation challenges (difference between human and animal models, biodistribution, scale-up, nanoparticle optimization)							
Weissig et al., 2014.	x	x	x	x	x	x	
Discussion on pharmacoeconomy and clinical data on AmB formulations							
Voltan et al., 2016.	x	x	x	x	x		
Only focus on antifungals, on targeted microorganisms and related indications and products under pre-clinical development							
Sosnik and Carcaboso, 2014.	x		x	x	x	x	x
Only focus on pediatric diseases, and mainly on cancer							
Fernandez-Garcia et al., 2017.	x	x	x	x	x	x	x
Emphasize on the type of drug delivery system and the effect of particle size on efficacy, toxicity and biodistribution							

References

Hafner A., Lovrić J., Lakoš G.P., Pepić I. Nanotherapeutics in the EU: an overview on current state and future directions. Int J Nanomedicine 2014;9:1005–23. https://doi.org/10.2147/IJN.S55359.

Bekersky I., Fielding R.M., Buell D., Lawrence I. Lipid-based amphotericin B formulations: from animals to man. Pharm Sci Technol Today 1999;2:230–6. https://doi.org/10.1016/s1461-5347(99)00160-1.

Allen T.M., Cullis P.R. Liposomal drug delivery systems: from concept to clinical applications. Adv Drug Deliv Rev 2013;65:36–48. https://doi.org/10.1016/j.addr.2012.09.037.

Bobo D., Robinson K.J., Islam J., Thurecht K.J., Corrie S.R. Nanoparticle-Based Medicines: A Review of FDA-Approved Materials and Clinical Trials to Date. Pharm Res 2016;33:2373–87. https://doi.org/10.1007/s11095-016-1958-5.

Anselmo A.C., Mitragotri S. Nanoparticles in the clinic: An update. Bioeng Transl Med 2019;4:e10143. https://doi.org/10.1002/btm2.10143.

Anselmo A.C., Mitragotri S. Nanoparticles in the clinic. Bioeng Transl Med 2016;1:10–29. https://doi.org/10.1002/btm2.10003.

Weissig V., Pettinger T.K., Murdock N. Nanopharmaceuticals (part 1): products on the market. Int J Nanomedicine 2014;9:4357–73. https://doi.org/10.2147/IJN.S46900.

Voltan A.R., Quindós G., Alarcón K.P.M., Fusco-Almeida A.M., Mendes-Giannini M.J.S., Chorilli M. Fungal diseases: could nanostructured drug delivery systems be a novel paradigm for therapy? Int J Nanomedicine 2016;11:3715–30. https://doi.org/10.2147/IJN.S93105.

Sosnik A., Carcaboso A.M. Nanomedicines in the future of pediatric therapy. Adv Drug Deliv Rev 2014;73:140–61. https://doi.org/10.1016/j.addr.2014.05.004.

Fernandez-Garcia R., de Pablo E., Ballesteros M.P., Serrano D.R. Unmet clinical needs in the treatment of systemic fungal infections: The role of amphotericin B and drug targeting. Int J Pharm 2017;525:139–48. https://doi.org/10.1016/j.ijpharm.2017.04.013.
